# I536T variant of *RBM20* affects splicing of cardiac structural proteins that are causative for developing dilated cardiomyopathy

**DOI:** 10.1007/s00109-022-02262-8

**Published:** 2022-10-05

**Authors:** Takuma Yamamoto, Rie Sano, Aya Miura, Mai Imasaka, Yoshiro Naito, Minori Nishiguchi, Kensuke Ihara, Naruhito Otani, Yoshihiko Kominato, Masaki Ohmuraya, Hidehito Kuroyanagi, Hajime Nishio

**Affiliations:** 1 Department of Legal Medicine, Hyogo Medical University, 1-1 Mukogawa-cho, Nishinomiya, Hyogo 663-8501 Japan; 2 Department of Legal Medicine, Gunma University Graduate School of Medicine, Gunma, 371-8511 Japan; 3 Department of Genetics, Hyogo Medical University, Hyogo, 663-8501 Japan; 4 Department of Cardiovascular and Renal Medicine, Hyogo Medical University, Hyogo, 663-8501 Japan; 5 Department of Bio-Informational Pharmacology, Medical Research Institute, Tokyo Medical and Dental University, Tokyo, 113-8510 Japan; 6 Department of Public Health, Hyogo Medical University, Hyogo, 663-8501 Japan; 7 Department of Biochemistry, Graduate School of Medicine, University of the Ryukyus, Okinawa, 903-0215 Japan

**Keywords:** Sudden death, *RBM20*, Cardiomyopathy, Alternative splicing, Postmortem examination

## Abstract

**Abstract:**

*RBM20* is one of the genes predisposing to dilated cardiomyopathy (DCM). Variants in the RS domain have been reported in many DCM patients, but the pathogenicity of variants within the RNA-recognition motif remains unknown. Two human patients with the I536T-*RBM20* variant without an apparent DCM phenotype were identified in sudden death cohorts. A splicing reporter assay was performed, and an I538T knock-in mouse model (*Rbm20*^*I538T*^) was generated to determine the significance of this variant. The reporter assay demonstrated that the human I536T variant affected the *TTN* splicing pattern compared to wild-type. In the mouse experiments, *Rbm20*^*I538T*^ mice showed different splicing patterns in *Ttn*, *Ldb3*, *Camk2d*, and *Ryr2*. The expressions of *Casq1*, *Mybpc2*, and *Myot* were upregulated in *Rbm20*^*I538T*^ mice, but *Rbm20*^*I538T*^ mice showed neither DCM nor cardiac dysfunction on histopathological examination and ultrasound echocardiography. The I536T-*RBM20* (I538T-*Rbm20*) variant changes gene splicing and affects gene expression, but the splicing and expression changes in *Ttn* and Ca handling genes such as *Casq1*, *Camk2d*, and *Ryr2* do not cause DCM morphology in the mouse model.

**Key messages:**

• Two human patients with the I536T-*RBM20* variant without a DCM phenotype were identified.

• A splicing reporter assay demonstrated that the variant affected the *TTN* splicing.

• *Rbm20*^*I538T*^ mice showed neither DCM nor cardiac dysfunction.

• *Rbm20*^*I538T*^ mice showed different splicing patterns and the gene expressions.

**Supplementary Information:**

The online version contains supplementary material available at 10.1007/s00109-022-02262-8.

## Introduction

Sudden unexpected death in a young adult is a rare event, but elucidating its mechanisms is one of the most important issues in medical science. Recently, genetic analysis has been improved and adopted in the field of postmortem examination, and there are many reports and studies in which postmortem genetic analysis uncovered the mechanism of autopsy-negative sudden death [1–4]. Most studies used target sequencing and exome sequencing with next-generation sequencing. However, problems arise when comprehensive exome sequencing detects many genetic variants, and the interpretation becomes difficult [5]. The mechanism at the molecular biological level is important.

*RBM20*, encoding RNA Binding Motif Protein-20 (RBM20), is the regulator of heart-specific pre-mRNA splicing [6]. Mutations in this gene were identified in human-dilated cardiomyopathy (DCM, MIM613172) patients [7]. Since these first patients with *RBM20* variants [7], several dozen variants have been reported so far [8], most of which were associated with DCM phenotypes.

One of the target genes of RBM20 is *TTN* [6, 9–11]. The protein product of the *TTN* gene, titin, composes part of the sarcomere and also acts as a molecular spring. It has the largest number of exons in humans and has various types of splice variants. The isoforms of cardiac types include the shorter N2B and the longer N2BA isoforms, and that of the skeletal muscle types includes the N2A isoform. Abnormal splicing of *TTN* caused by *RBM20* variants is considered to contribute to DCM phenotypes. In the mouse model with *RBM20* pathogenic variants, an abnormal giant N2BA isoform, the N2BA-G isoform, is predominant. It results from inclusion of apparently all the alternatively spliced exons of Ig and PEVK (proline, glutamate, valine, lysine) regions, all of which are not included in the normal cardiac isoforms. This giant molecular spring provides reduced passive stiffness to the cardiomyocyte, contributing to DCM [12].

The mutation hotspot region of *RBM20* is within the RS domain [8, 13], among which P633L, R634Q, R634W, R634L, S635A, R636S, R636C, R636H, S637G, and P638L are within the RSRSP stretch. In contrast, few *RBM20* disease variants have been found within the RNA-recognition motif (RRM) domain. Of the *RBM20-*related DCMs, the variants in RS-domain show familial DCM, whereas a single example of a V535I variant was found in a sporadic DCM patient [14]. The G583C variant was also reported in a hypertrophic cardiomyopathy (HCM) patient [15]. Recently, we also reported a sudden death patient with a heterozygous I536T variant, situated in the strictly conserved RRM region [16]. This sudden death patient with the I536T variant did not show DCM morphologically, and thus, the effect of I536T-*RBM20* on the cardiac function is controversial.

In this study, molecular biological analysis was performed by using a splicing reporter assay, and then an I538T knock-in (KI) mouse model (*Rbm20*^*I538T*^) was generated to determine the relevance of this variant.

## Materials and methods

### Editorial policies and ethical considerations

All research was performed in accordance with the guidelines for human genome analysis in Japan, and the study protocol was approved by the Ethics Committee of Hyogo Medical University (0358) and Gunma University Graduate School of Medicine (2017–015). Written, informed consent for genetic analysis was obtained from the relatives. All animal experiments were performed with the approval of the Hyogo Medical University Institutional Animal Care and Use Committee (18–078) and the ARRIVE guideline.

### Identification of RBM20 variants in human sudden death patients

Next-generation sequencing was used to screen sudden death patients for variants in exons and exon–intron boundaries of *RBM20*. The variants detected were confirmed by Sanger sequencing, as described elsewhere.

### Plasmid construction

The *TTNE50-E51E218-E219-EGFP/mCherry* minigene reporter plasmid was constructed as previously described [9]. Expression plasmids for human *RBM20* protein pRP[Exp]-Puro-CAG > hRBM20[NM_001134363.3] were purchased from VectorBuilder Inc (Chicago, IL). Using overlapping PCR mutagenesis, the mutations of c.T1607C were inserted into the wild-type cDNA in constructs of pRP-hRBM20I536T. For all constructs, sequencing was performed over the entire region of the amplified sequences. Plasmid DNA was purified using a HiSpeed® Plasmid Maxi Kit (Qiagen, Venlo, Netherland).

### Cell culture and transfection

HeLa cells were cultured in DMEM Medium (FUJIFILM Wako Pure Chemical, Osaka, Japan) supplemented with 10% fetal bovine serum at 37 °C with 5% CO_2_. The *TTN* reporter minigene and the expression vectors were transiently co-transfected in a 1:4 mixture into Hela cells using Lipofectamine (Thermo Fisher Scientific, Waltham, MA) in accordance with the manufacturer’s protocol. Fluorescence images of fluorescent proteins were acquired 24 h after transfection using EVOS (Thermo Fisher Scientific), and then the cells were harvested for total RNA preparation and RT-PCR analysis.

### Total RNA extraction and RT-PCR of the reporter assay

Total RNAs from HeLa cells involved in transient transfection experiments were extracted using the RNeasy mini kit (Qiagen). Complementary DNAs (cDNAs) were prepared with the SuperScript III First-Strand Synthesis System (Thermo Fisher Scientific) using 1 µg of RNA with a random primer, in accordance with the manufacturer’s protocol. Semi-quantitative PCRs were performed using PrimeStarGXL (Takara Bio Inc., Shiga, Japan), and the PCR products were analyzed using the Bioanalyzer 2100 Expert with DNA7500 Kit (Agilent Technologies Inc., Santa Clara, CA). Primers and conditions of RT-PCR were described previously [9].

### Animal procedures

Mice were kept in a temperature-controlled facility under specific-pathogen-free conditions with ad libitum access to food and water in a 12-h light/dark cycle. C57BL/6 J mice were purchased from CLEA Japan (Tokyo, Japan).

### Preparation of Cas9/gRNA complex and ssDNA

The gRNA was designed using CRISPRdirect (https://crispr.dbcls.jp) to target exon 6 of *Rbm20*. The gRNA sequence was as follows: Rbm20 gRNA 5′-GGAGAATGACGTCATTAACC-3′. The corresponding crRNA (5′-GGAGAAUGACGUCAUUAACCGUUUUAGAGCUAUGCU-3′), tracrRNA (#1,072,533), and Cas9 nuclease were purchased from Integrated DNA Technologies (IDT, Coralville, IA). The crRNA and tracrRNA were annealed and complexed with Cas9 nuclease according to the manufacturer’s instructions.

Single-strand DNA (ssDNA) with c.T1613C mutation was ordered as PAGE Ultramer from IDT. The ssDNA sequence was as follows: Rbm20 ssDNA 5′-GCACATCTGCAATCTCCCGGAGGGCAGCTGCACGGAGAATGACGTCACTAACCTGGGGCTGCCCTTTGGCAAGGTCACTAATTACATCCTCATGAAGTCA-3′.

### Electroporation

Male and female C57BL/6 mice (CLEA Japan) were used as sperm and oocyte donors. Female mice were superovulated, and then zygotes were prepared by in vitro fertilization. They were cultured in KSOM medium (ARK Resource, Kumamoto, Japan) before and after electroporation. Electroporation was performed using a 1-mm gap electrode (CUY501P1-1.5) and a super electroporator NEPA21 (Nepa Gene, Chiba, Japan). Zygotes were washed with Opti-MEM (Thermo Fisher Scientific) and then placed in the electrode gap filled with 5 µl of Opti-MEM solution containing 200 ng/µL Cas9 protein, 6 pmol/µL gRNA (crRNA/tracrRNA complex), and 10 pmol/µL ssDNA. They were cultured in KSOM medium overnight, and surviving 2-cell-stage embryos were transferred to the oviducts of pseudopregnant Jcl:ICR female mice (CLEA Japan).

### Genomic DNA extraction, genotyping, and sequencing

Genomic DNA was extracted from F0, F1, and F2 mice. PCR was performed using Taq DNA polymerase (Greiner Bio-One, Kremsmünster, Austria). The primer sequences are shown in Supplementary Material, Table S1. The PCR products were purified using the Gel/PCR Extraction Kit (NIPPON Genetics, Tokyo, Japan). The purified PCR products were sequenced using the BigDye Terminator v3.1 Cycle Sequencing Kit (Thermo Fisher Scientific) through an Applied Biosystems 3500xL Genetic Analyzer (Thermo Fisher Scientific), or restriction enzyme digestion was performed by RspRS II enzyme (Takara Bio Inc.), according to the manufacturer’s instructions. In some cases, PCR products were cloned into TA vector (Promega, Madison, WI) before sequencing.

### Total RNA extraction and RNA sequencing

After euthanasia, total RNAs from mouse hearts at the ages of 1, 12, and 36 weeks were extracted using the RNeasy Plus Universal Mini Kit (Qiagen). Nine representative libraries were generated for the nine groups (wild-type, heterozygote, and homozygote of 1 week, 12 weeks, and 36 weeks) by RNA-seq with SMART-seq v4 Ultra Low Input RNA kit for sequencing (Takara Bio Inc.) and Nextera XT DNA Library Prep Kit (Illumina, San Diego, CA) and sequenced on a NovaSeq 6000 instrument (Illumina).

### Bioinformatic analysis

Splicing analysis with Percentage Spliced In (PSI) was performed as described previously [16]. Differential gene expression analysis was performed using StrandNGS (Strand Life Sciences Pvt. Ltd., Bangalore, India). The data discussed in this publication have been deposited in NCBI’s Gene Expression Omnibus and are accessible through GEO Series accession number GSE201018.

### Splicing analysis

Complementary DNA was synthesized from total RNA (1 µg) using a Prime-Script RT PCR Kit (Takara Bio Inc.), according to the manufacturer’s instructions. The confirmation of the quantification of the splicing variant was performed as previously described [16]. In detail, total RNA was reverse transcribed to cDNA, and PCR amplification was performed using PrimeSTAR Max Premix (2) (Takara Bio Inc.), with the primers shown in Supplementary Material, Table S2. Each splicing variant of the PCR product was shown using an Agilent 2100 Bioanalyzer performed with a DNA 1000 LabChip Kit and High sensitivity LabChip Kit (Agilent Technologies Inc.). For semi-quantification, the proportions of splicing variant were calculated using the following formula: proportion = amount of the one product/ (amount of the one product + amount of the other product).

### Quantitative real-time PCR

Quantitative real-time PCR (qRT-PCR) was performed using TB Green® Premix Ex Taq™ II (Takara Bio Inc.) and a Thermal Cycler Dice Real Time System (Takara Bio Inc.). Sequences of the PCR primers used are presented in Supplementary Material, Table S3. Expression levels were normalized with *Ttn* exon50 or *Gapdh*. Statistical significance was assessed by Prism9.

### Histopathology

For the histological analyses, heart tissue at the age of 12 weeks was fixed in paraformaldehyde, 4% in phosphate-buffered saline, embedded in paraffin, sectioned, and subjected to hematoxylin–eosin staining and Masson’s trichrome staining. Stained sections were photographed using Virtual Slide System VS120 (Olympus Scientific Solutions, Tokyo, Japan).

### Cardiovascular phenotyping

Ultrasound echocardiography (UCG) was performed in a blinded manner. In detail, mice at the age of 12 weeks were anesthetized with isoflurane and placed on a heated platform. Cardiac functions, such as left ventricular fractional shortening (LVFS), end-diastolic dimension (LVDd), and diastolic wall thickness of the LV posterior wall (LVPWth), were assessed using a high-frequency ultrasound system (Vevo 2100 with MS400, FUJIFILM VisualSonics, Inc., Toronto, Canada). A two-dimensional parasternal short-axis view was obtained at the levels of the papillary muscles, and M-mode tracings were recorded.

## Results

### Human cases of RBM20 I536T presented sudden death without apparent DCM

**Fig. 1:**
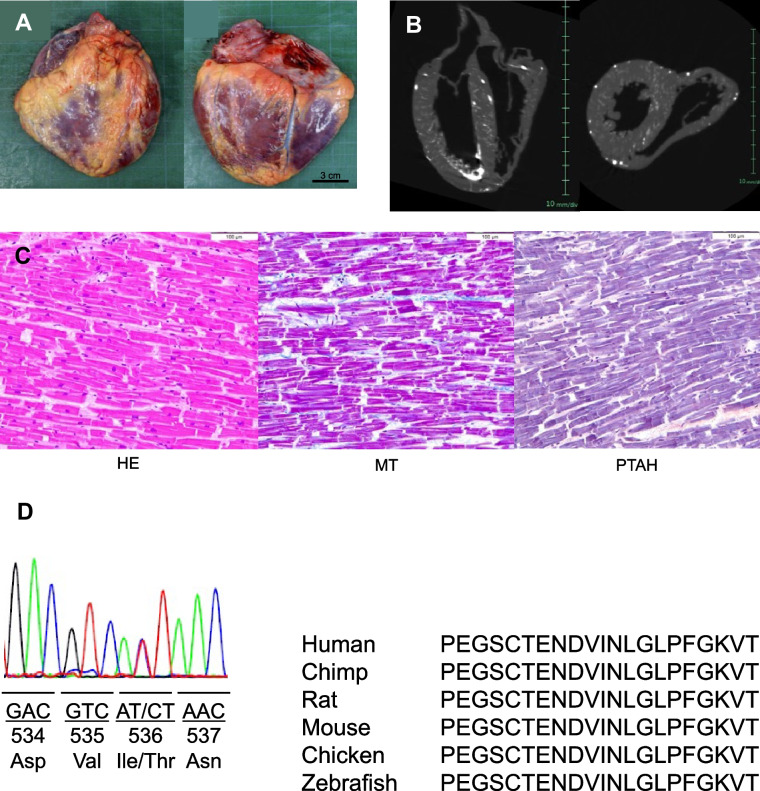
Human sudden death case. **A**, **B** Macroscopic examination of heart **A** and computed tomography image **B**. No cardiac dilatation is observed DCM. **C** Microscopic examination shows disarray and diffuse mild fibrosis in the heart. **D** Postmortem genetic analyses show the I536T-*RBM20* variant. It is conserved among the species

Two Japanese sudden death patients who possessed the I536T-*RBM20* variant were identified. The first patient was reported previously [16]. The second patient was a male in his 20 s, who had no history of arrhythmia (Supplementary Material, Fig. S1) or collapse. He was found dead in bed during sleep. He had no family history of sudden death or arrhythmia. His weight was 58 kg, and his height was 167 cm. The heart weighed 320 g (Fig. 1A). No cardiac dilatation was observed (Fig. 1B). The coronary arteries showed no stenoses. The left and right lungs weighed 610 g and 640 g, respectively, and the edematous brain weighed 1670 g. Microscopic examination showed disarray and diffuse mild fibrosis in the heart (Fig. 1C).

Postmortem genetic analyses showed that both patients had the I536T-*RBM20* variant (Fig. 1D). The familial analyses were not allowed. The residue was conserved among the species and was located in the RRM region. In silico prediction programs suggested that this variant is likely pathogenic: probably damaging by PolyPhen-2 and Deleterious by SIFT.

### Human RBM20 I536T mutation affects TTN splicing in the splicing reporter assay

**Fig. 2:**
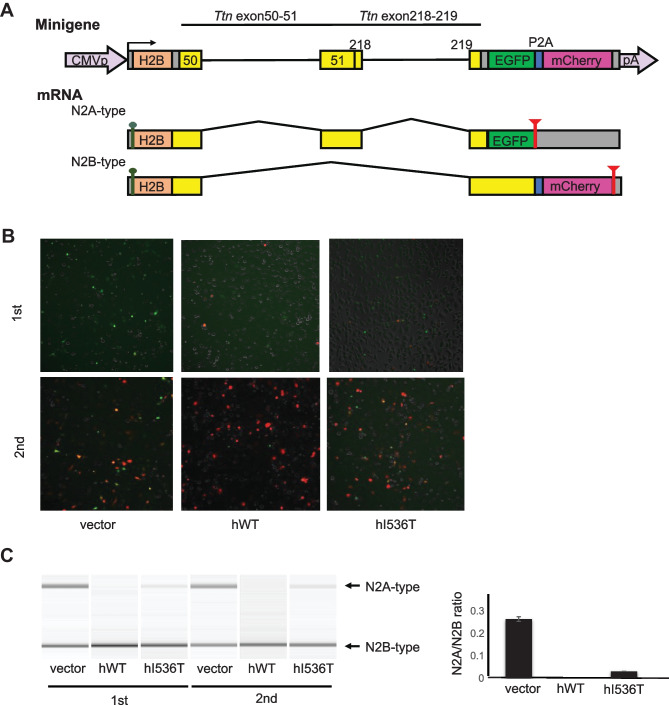
Dichromatic fluorescence alternative splicing reporter for *Ttn* to monitor the *RBM20* activity in splicing regulation. **A** Schematic representation of the *Ttn* reporter minigene TtnE50-E51E218-E219-EGFP/mCherry (top) and mRNAs derived from it (bottom). Two genomic fragments *Ttn* E50-E51 and E218-E219 are inserted between human histone H2B cDNA and the EGFP/mCherry cassette. Expression of E51E218EGFP and ΔE51E218-mCherry indicates inclusion and skipping of a chimeric exon E51E218, respectively. **B** Microphotographs of HeLa cells co-transfected with the fluorescence *Ttn* splicing reporter minigene and an empty vector or an expression vector for the wild-type or mutant *RBM20* protein. Fluorescence of EGFP and mCherry is pseudo-colored in green and magenta, respectively. The experiment was performed twice. **C** RT-PCR analysis of the *TTN* splicing reporter minigene co-expressed with an empty vector or an expression vector for the wild-type or mutant *RBM20* protein in HeLa cells. Representative gel-like presentations are shown. The ratio of N2A/N2B is shown. The N2A type is not detected with the wild-type RBM20, whereas it is still detected with the I536T mutant vector

To evaluate the effect of the *RBM20* I536T mutation on the splicing of the *TTN* gene, in vitro assays were performed using a dichromatic *Ttn* alternative splicing reporter minigene. Human *RBM20* wild-type, as well as I536T mutant, vectors were co-transfected with the reporter minigene vector into Hela cells. Green fluorescent protein (EGFP) is expressed from the product containing the fused exon E51E218, and red fluorescent protein (mCherry) is translated from the E51E218-skipped mRNA, corresponding to the splicing patterns of the N2A and N2B types, respectively (Fig. 2A).

In Hela cells co-transfected with an empty vector, fluorescence of both N2A-type EGFP and N2B-type mCherry was detected (Fig. 2B). Co-expression of human wild-type *RBM20* promoted the expression of N2B-type mCherry and suppressed expression of N2A-type EGFP (Fig. 2B, middle panel). On the other hand, co-expression of the human I536T mutant vector less efficiently promoted expression of N2B-type mCherry, and expression of N2A-type EGFP still remained (Fig. 2B, right panel). In addition, the splicing pattern of mRNAs derived from the minigene was examined by using semi-quantitative RT-PCR. The remaining expression of N2A type by transfection of the I536T mutant vector was confirmed by electrophoresis, and N2A/N2B ratio was increased in the I536T mutant vector (Fig. 2C). These results suggested that human *RBM20* I536T affects the splicing of *TTN*.

### Generation of the KI mice

Using the CRISPR/Cas9 system, KI mice harboring the c.T1613C (p.I538T) point mutation in *Rbm20*, which corresponded to human c.T1607C (p.I536T), were generated (Supplementary Material, Fig. S2 and Fig. S3). Through electroporation of the Cas9/gRNA complex and donor ssDNA into zygotes, 36 founder (F0) mice were born. Of them, 14 mice (39%) had the intended point mutation. These F0 mice were crossed with C57BL/6 J mice (CLEA Japan), and heterozygous F1 mice, which were confirmed by Sanger’s sequencing, were obtained.

The KI mice were born according to Mendel’s law and were all healthy, fertile, and indistinguishable from their wild-type littermates.

### Rbm20 KI mice showed abnormal splicing

**Fig. 3:**
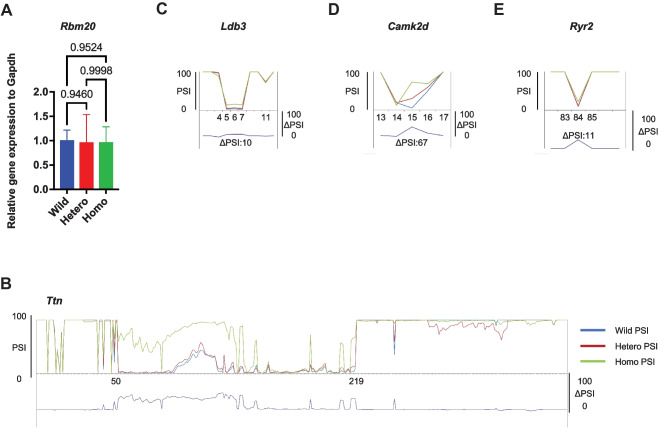
*Rbm20*.^*I538T*^ knock-in mice show abnormal splicing in the heart (RNA-seq analysis). **A** *Rbm20* RNA levels normalized to *Gapdh* and wild-type levels are not different on qRT-PCR in the heterozygote and the homozygote. *n* = 3 each. **B**–**E** The average PSI of 3 mice per genotype is shown (wild-type in blue, heterozygote in red, homozygote in green), and the resulting ΔPSIs (wild-type vs. homozygotes) show abnormal exon inclusion or exclusion in *Ttn* **B**, *Ldb3* **C**, *Camk2d* **D**, and *Ryr2* **E**

To exclude the effect of altered *Rbm20* expression on splicing of other genes, qRT-PCR was performed, and there were no differences in *Rbm20* expression among the three genotypes (Fig. 3A).

To determine whether the *RBM20* variant affects the splicing of the target genes such as *Ttn*, RNA-seq was performed on heart samples from three genetic types of each age. Splicing analysis with PSI showed a higher percentage for the *Ttn* exons encoding I band region (ΔPSI of approximately 60% between wild-type and homozygous type), and various abnormal splicing patterns in *Ldb3*, *Camk2d*, and *Ryr2* were also detected (Fig. 3B–E), which were also detected in other KI mice [17].

**Fig. 4:**
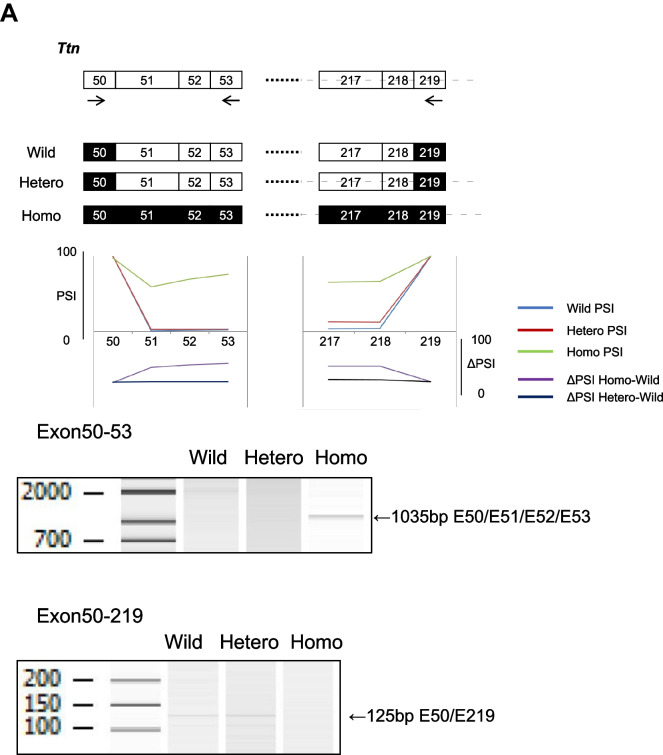

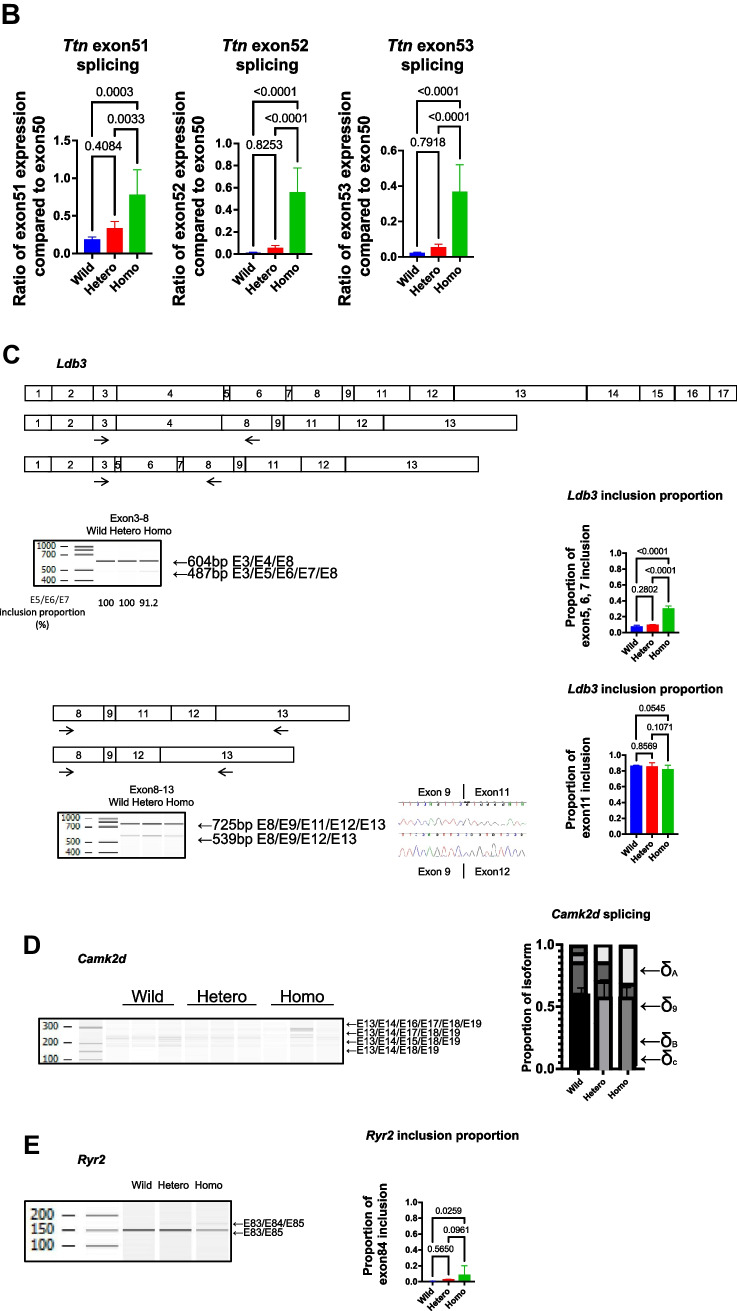
*Rbm20*^*I538T*^ knock-in mice have abnormal splicing in the heart (RT-PCR validation). **A** Schematic representation of the *Ttn* isoform and the re-indication of the RNA-seq data are shown. The bands of RT-PCR analysis are detected in the homozygote (left, exon50-53) and in the wild-type and heterozygote (right, exon50-219). **B** Relative expression of *Ttn* exons 51, 52, and 53 compared to exon50 shows that these exons are more included in the homozygote than the wild-type and the heterozygote. *n* = 6 each. **C** Schematic representation of the *Ldb3* isoforms. The RT-PCR analysis shows that exon4 (cardiac isoform, 604 bp) is included in all genotypes, whereas exons 5, 6, and 7 (skeletal isoform, 487 bp) are included predominantly in the homozygote, which was hardly detected in the wild-type or the heterozygote. The inclusion proportion is significantly different among the genotypes. *n* = 6 each. The RT-PCR analyses of exon11 inclusion/exclusion are shown, and there is no significant difference among the genotypes. *n* = 6 each. **D** The RT-PCR analysis of *Camk2d* mRNAs shows isoform differences among the genotypes. The graph shows the proportion of each isoform. **E** The RT-PCR analysis of *Ryr2* mRNAs shows abnormal 24-bp exon inclusion in the homozygote. There is significant difference among the genotypes. *n* = 3 each. Error bars indicate 95% confidence interval, Tukey’s multiple comparison test

To validate this abnormal splicing, RT-PCR was performed and confirmed the abnormal splicing patterns of these genes. In addition, qRT-PCR showed that inclusion of *Ttn* exons 51, 52, and 53 was increased in the homozygous type compared to the wild-type (Fig. 4A, B).

Exon 4 of *Ldb3* is included in the cardiac isoform, whereas exons 5, 6, and 7 are included instead in the skeletal isoform. PSI analysis and RT-PCR showed an abnormal splicing pattern in the homozygotes, i.e., inclusion of exons 5, 6, and 7, in the cardiac tissue (Fig. 4C). To confirm the isoforms with exon 11 inclusion/exclusion, RT-PCR was performed from exon 8 to exon 13 of *Ldb3* and showed that the inclusion/exclusion ratio of exon 11 of *Ldb3* was not different among the three genotype groups (Fig. 4C).

*Camk2d* has several types of isoforms [18, 19]. PSI analysis and RT-PCR showed that the longer isoform including exons 13, 15, 16, and 17 (1A) increased in the homozygotes, whereas the shorter one including exons 13 and 17 (2C) was predominant in the wild-type (Fig. 4D).

The homozygous type included an additional 24-bp exon in the *RyR2* gene, which was confirmed by RT-PCR (Fig. 4E).

### Three mRNAs were upregulated in the homozygous Rbm20 KI mice

**Fig. 5:**
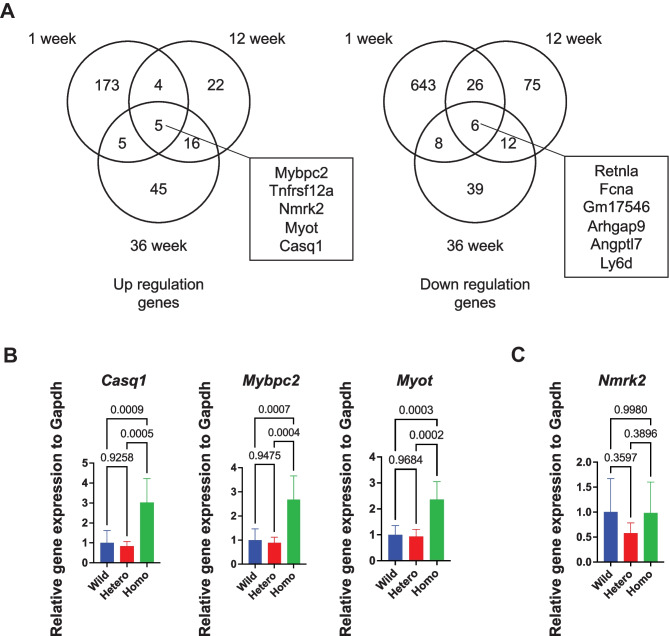
Gene expression profile analysis with RNA-seq data. **A** Venn diagram analysis of differentially expressed genes at 3 weeks (left, upregulated genes; right, downregulated genes). **B** qRT-PCR analysis of upregulated genes at the age of 12 weeks. *n* = 6 each. **C** qRT-PCR analysis of *Nmrk2* genes at the age of 12 weeks. *n* = 6 each. Error bars indicate 95% confidence interval, Tukey’s multiple comparison test

To compare the gene expression profiles in *Rbm20* KI mice, RNA-seq from the heart was performed at 1, 12, and 36 weeks of age. A total of 1079 differentially expressed genes (DEGs) between the WT and *Rbm20* KI mice (270 upregulated and 809 downregulated genes) were identified (Fig. 5A). Of them, 5 genes were upregulated at every stage of age, including *Casq1*, *Mybpc2*, and *Myot*, which were related to heart functions. These three upregulated genes were then examined, and qRT-PCR showed that the expressions of *Casq1*, *Mybpc2*, and *Myot* were significantly increased in the homozygotes (Fig. 5B). *Nmrk2*, which was highly expressed in DCM [20], was also upregulated in the RNA-seq analysis, but qRT-PCR analysis showed no significant difference (Fig. 5C).

### Rbm20 KI mice did not show sudden death or a cardiac phenotype

**Fig. 6:**
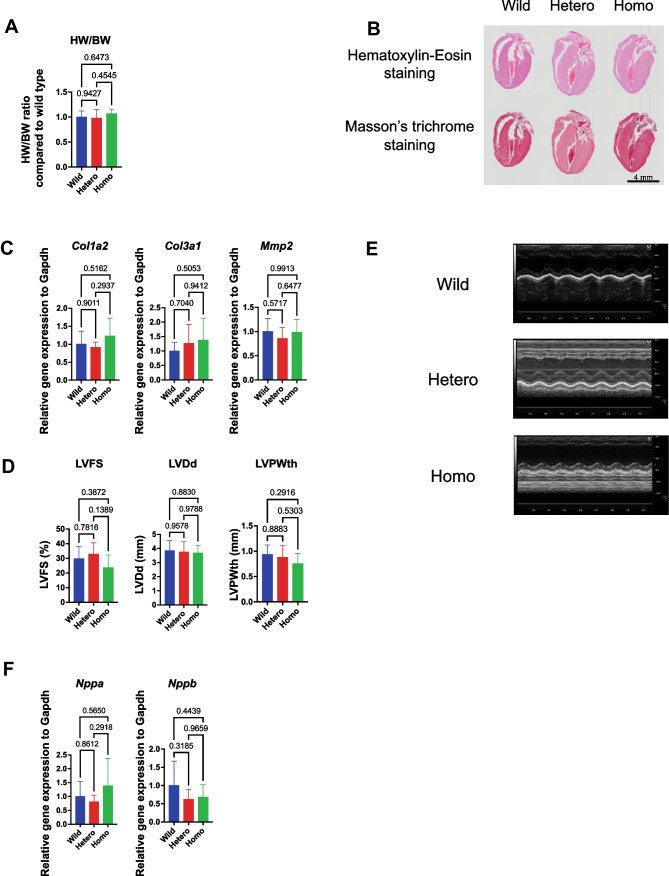
*Rbm20*^I538T^ knock-in mice do not show cardiac dysfunction. **A** Heart/body weight ratio at the age of 12 weeks. *n* = 6 each. **B** Representative images of H&E staining and Masson’s trichrome staining at the age of 12 weeks. Scale bars, 4 mm. **C** qRT-PCR analysis of genes related to cardiac fibrosis at the age of 12 weeks. *n* = 6 each. **D** Measured values of LVFS, LVDd, and LVPWth by UCG at the age of 12 weeks. *n* = 6 each. **E** Representative images of UCG at the age of 12 weeks. **F** Gene expression of *Nppa* and *Nppb* in the heart assessed by qRT-PCR at the age of 12 weeks. *n* = 6 each. Error bars indicate 95% confidence interval. Tukey’s multiple comparison test

None of the *Rbm20* KI heterozygous or homozygous mice experienced sudden death until the age of 36 weeks (*n* = 6), which is different from the human sudden death cases with the same *RBM20* variant. Heart weight was not different among genotypes (Fig. 6A). Pathological examination showed no cardiac dilation and subendocardial fibrosis (Fig. 6B). The expressions of *Col1a2*, *Col3a1*, and *Mmp2* mRNAs were not increased in the *Rbm20* KI mice (Fig. 6C), consistent with the lack of fibrosis.

To assess the cardiac phenotypes of the *Rbm20* mutant mice, UCG was performed. The UCG data showed no significant difference in LVFS, LVDd, and LVPWth (Fig. 6D, E). Indeed, LVFS of some of the homozygous mice seemed to be decreased, as shown in Fig. 6D, though there were no significant differences because of the small sample size. However, the relative expressions of *Nppa* and *Nppb*, which are biomarkers of heart failure, were not upregulated (Fig. 6F).

## Discussion

In the present study, two human cases of sudden death with I536T-*RBM20* were presented, and, using in vitro and in vivo analyses, it was demonstrated that this variant affected alternative splicing and gene expression without an apparent cardiac phenotype.

There are three main issues to be addressed. First, patient 2 presented with sudden death, and genetic analysis showed I536T-*RBM20*, but splicing analysis was not performed, and the splicing effect was unknown. Second, patient 1, described elsewhere [16], had an asymptomatic genetic background of myotonic dystrophy type 1 (DM1) with expanded CTG repeats in the *DMPK* gene, in addition to I536T-*RBM20*. Because both genetic predispositions affect exon splicing [21], it was not possible to determine with any certainty whether the abnormal splicing of patient 1 was caused by I536T-*RBM20* alone [16]. Third, we have no doubt that *RBM20* is one of the important causative genes of DCM [7], but the I536T-*RBM20* variant has never been reported, and the exact influence of this variant on DCM is still unknown. One goal of this study was to uncover the splicing effect in humans by using a reporter assay, and another major goal was to perform an analysis using a KI mouse model.

First, the effect of the I536T variant on human *TTN* splicing was surveyed. In patient 1, it was possible to obtain a frozen sample of heart tissue and perform RNA-seq analysis to calculate PSI. The splicing pattern of *TTN* in patient 1 was not the same as that in the control sample [16]. As for patient 2, because it was not possible to obtain a frozen sample, a reporter assay was performed as an alternative. The reporter assay showed abnormal *Ttn* splicing, which was consistent with the cardiac sample from patient 1. These results of the I536T variant were similar to other reports that abnormal *TTN* splicing was detected in patients with cardiac phenotypes who have mutations in *RBM20* [11, 22, 23], indicating that the I536T variant caused aberrant alternative splicing even in non-DCM patients.

Next, a KI mouse model of I538T-*Rbm20* was generated. There have been several types of animal models of *RBM20* cardiomyopathy [6, 17, 24–30]. Knock-out (KO)-model mice and rats showed abnormal splicing with only mild DCM phenotypes [6, 17, 24, 26, 27, 29]. KI mice and pigs with mutations in the RS region, such as R636S, S637A, and S639G, well mimicked human DCM phenotypes [17, 28, 30]. On the other hand, an RRM domain-deletion mouse model also showed the abnormal splicing in *Ttn*, *Camk2d*, and *Ldb3*, but did not show the DCM phenotype, such as LV chamber dilatation and systolic dysfunction [25].

In the present study, *Rbm20*^*I538T/I538T*^ mice showed abnormal splicing in *Ttn*, *Ldb3*, *Camk2d*, and *Ryr2*. The splicing in *Camk2d* showed change to the longer isoform of *Camk2d-1A*, and *Ryr2* included the additional 24-bp exon. These two genes are related to Ca^2+^ handling [11, 26, 27], which induces cardiac arrhythmias. The shift in CamkIIδ isoforms largely underlies the Ca^2+^ handling defects in another KO mouse model of splicing factors [31]. RNA-seq analysis showed that the expressions of *Casq1*, *Mybpc2*, *Myot*, and *Nmrk2* increased during the animals’ lifetime (1 week to 36 weeks of age). The clinical significance of the increase in these genes remains unknown, but *Casq1*, *Mybpc2*, and *Myot* were also increased in the other studies [17], and upregulation of these genes was validated. CASQ is one of the calcium-binding proteins, and both isoforms, CASQ1 and CASQ2, interact with the ryanodine receptors calcium release channels [32]. Human patients with *RBM20* variants are susceptible to arrhythmias such as ventricular tachycardia, leading to cardiac transplantation, implantation of an implantable cardioverter-defibrillator, or sudden death [7, 14, 27, 33–36]. Ventricular arrhythmias caused by *RBM20*-associated abnormal splicing of ion channels genes such as *RYR2* were also reported [37]. Thus, it has not yet been precluded that *Rbm20*^*I538T/I538T*^ mice have susceptibility to arrhythmia.

On the contrary, *Rbm20*^*I538T/I538T*^ mice did not show DCM, although *RBM20* cardiomyopathy often shows DCM phenotypes. *Nmrk2*, which was highly expressed in DCM [20], showed no significant difference on qRT-PCR analysis, though it was upregulated in RNA-seq analysis. Other biomarkers of heart failure, *Nppa* and *Nppb*, were also not upregulated. Considering the results of biophysical and morphological examinations, heart failure did not happen in *Rbm20*^*I538T/I538T*^ mice. In this respect, the human I536T patient and I538T mice are not typical.

In addition, *Rbm20*^*I538T/I538T*^ mice showed abnormal *Ldb3* splicing of exons 3, 4, 5, and 6, but they did not show a significant difference in the exon 11 inclusion/exclusion ratio. Abnormal splicing of exons 3 to 6 was reported in *RBM20* cardiomyopathy [6], and that of exon 11 was reported in myotonic dystrophy [38]. Patient 1 showed abnormal splicing in exon 11 [16]. The present experiment explained that this abnormal exon 11 splicing was not caused by the *RBM20* variant but was possibly caused by myotonic dystrophy.

This study has some future implications and limitations. This variant has not been reported, but the fact that these two patients were encountered at two independent institutes, and they were not consanguineous, means that there are possibly more sudden death cases with this variant. Both patients had a heterozygous I536T variant, which was thought to have been inherited from one of the parents without a familial history of cardiomyopathy or sudden death, though familial genetic analysis was not permitted. Thus, just having this variant does not seem to cause sudden death, and an unknown factor seemed to be a trigger. *Rbm20*^*I538T/I538T*^ mice were generated, and it was demonstrated that *Rbm20*^*I538T/I538T*^ mice mimicked human I536T-*RBM20* heterozygous patients in morphological phenotype and abnormal splicing, but they differed in that they did not suffer sudden death. This mouse model will have a great advantage in further studies of elucidating mechanisms of sudden death. Patient 1 had another genetic background, myotonic dystrophy. We would like to generate a double-transgenic mouse with *Rbm20* and *Dmpk* in the future. Investigating the genetic background, as well as uncovering the trigger of sudden death, is an important issue to diagnose the cause of death from congenital diseases. On the other hand, molecular biological analyses and morphological investigations were performed, but electrocardiograms were not analyzed. Further examination would be necessary.

In conclusion, two sudden death cases of human I536T-*RBM20* variant were presented. Splicing analysis of patient 2 was not performed directly, but the reporter assay showed an abnormal *Ttn* splicing. The mouse model showed abnormal splicing and different gene expressions without sudden death or cardiac failure. These splicing and expression changes in *Ttn* and Ca handling genes do not cause DCM morphology.

## Supplementary Information

Below is the link to the electronic supplementary material.

Supplementary file1 (PPTX 830 KB)

## Data Availability

The data sets used and analyzed during the current study are available from the corresponding author upon request. The data discussed in this publication have been deposited in NCBI’s Gene Expression Omnibus and are accessible through GEO Series accession number GSE201018.
