# Supplementary material for: I536T variant of RBM20 affects splicing of cardiac structural proteins that are causative for developing dilated cardiomyopathy
Source: J Mol Med (Berl). 2022 Oct 5;100(12):1741–54. doi: 10.1007/s00109-022-02262-8 (PMC9691496; doi:10.1007/s00109-022-02262-8)
Supplement: Supplementary file 1 — Supplementary file1 (PPTX 830 KB) [file 109_2022_2262_MOESM1_ESM.pptx]

## Slide 1
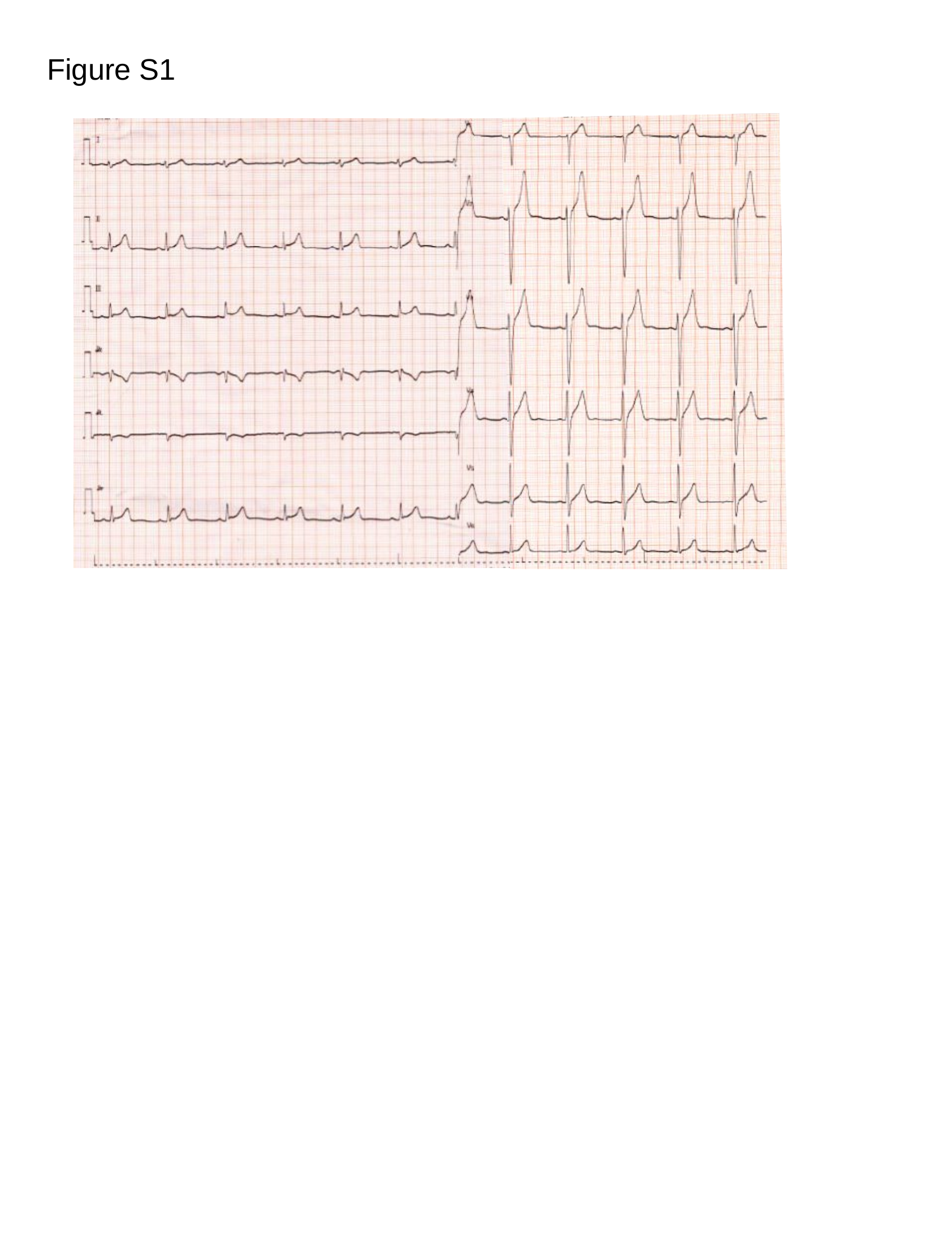

Figure S1

## Slide 2
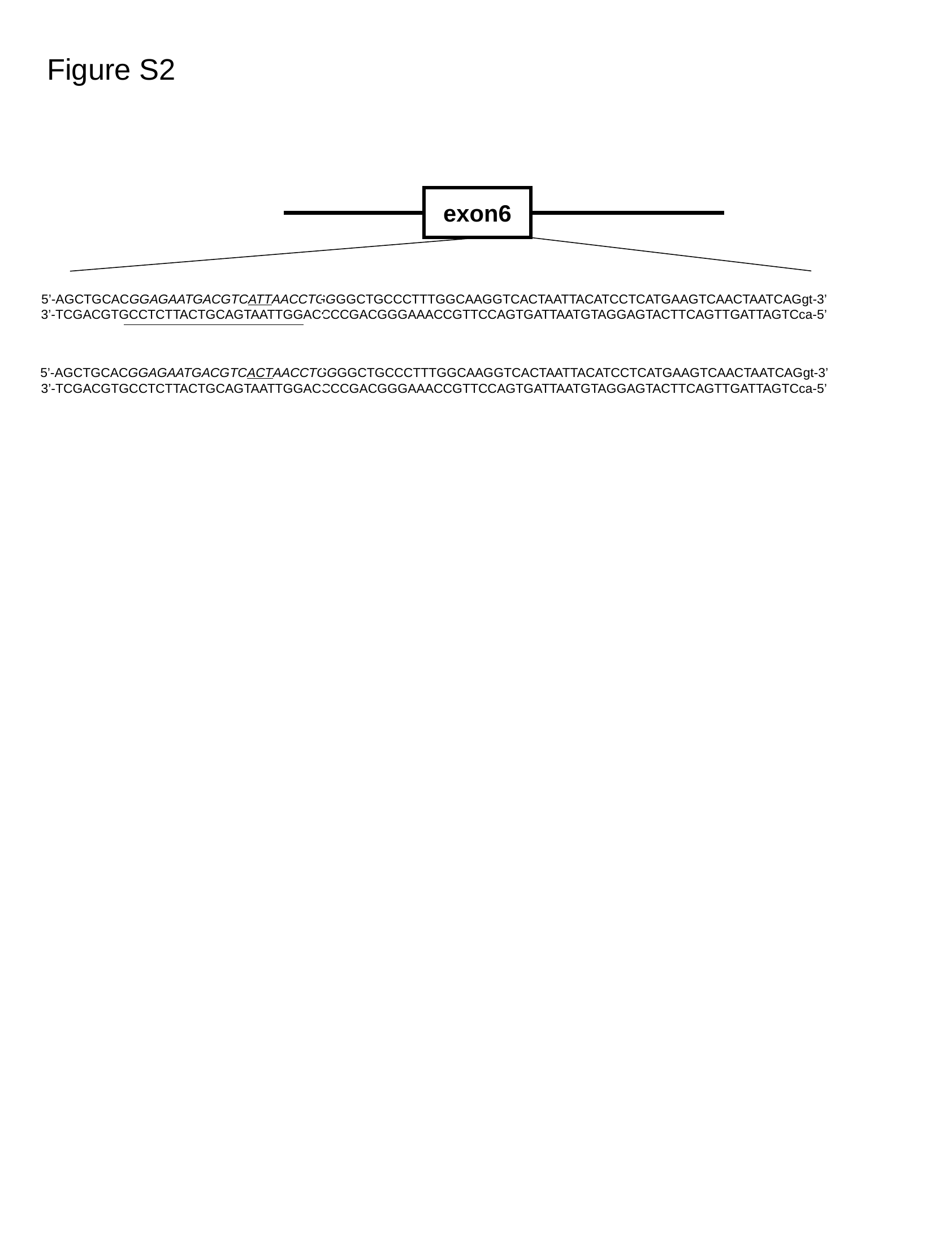

Figure S2
exon6
 5’-AGCTGCACGGAGAATGACGTCATTAACCTGGGGCTGCCCTTTGGCAAGGTCACTAATTACATCCTCATGAAGTCAACTAATCAGgt-3’
 3’-TCGACGTGCCTCTTACTGCAGTAATTGGACCCCGACGGGAAACCGTTCCAGTGATTAATGTAGGAGTACTTCAGTTGATTAGTCca-5’
Target sequence
PAM
 5’-AGCTGCACGGAGAATGACGTCACTAACCTGGGGCTGCCCTTTGGCAAGGTCACTAATTACATCCTCATGAAGTCAACTAATCAGgt-3’
 3’-TCGACGTGCCTCTTACTGCAGTAATTGGACCCCGACGGGAAACCGTTCCAGTGATTAATGTAGGAGTACTTCAGTTGATTAGTCca-5’

## Slide 3
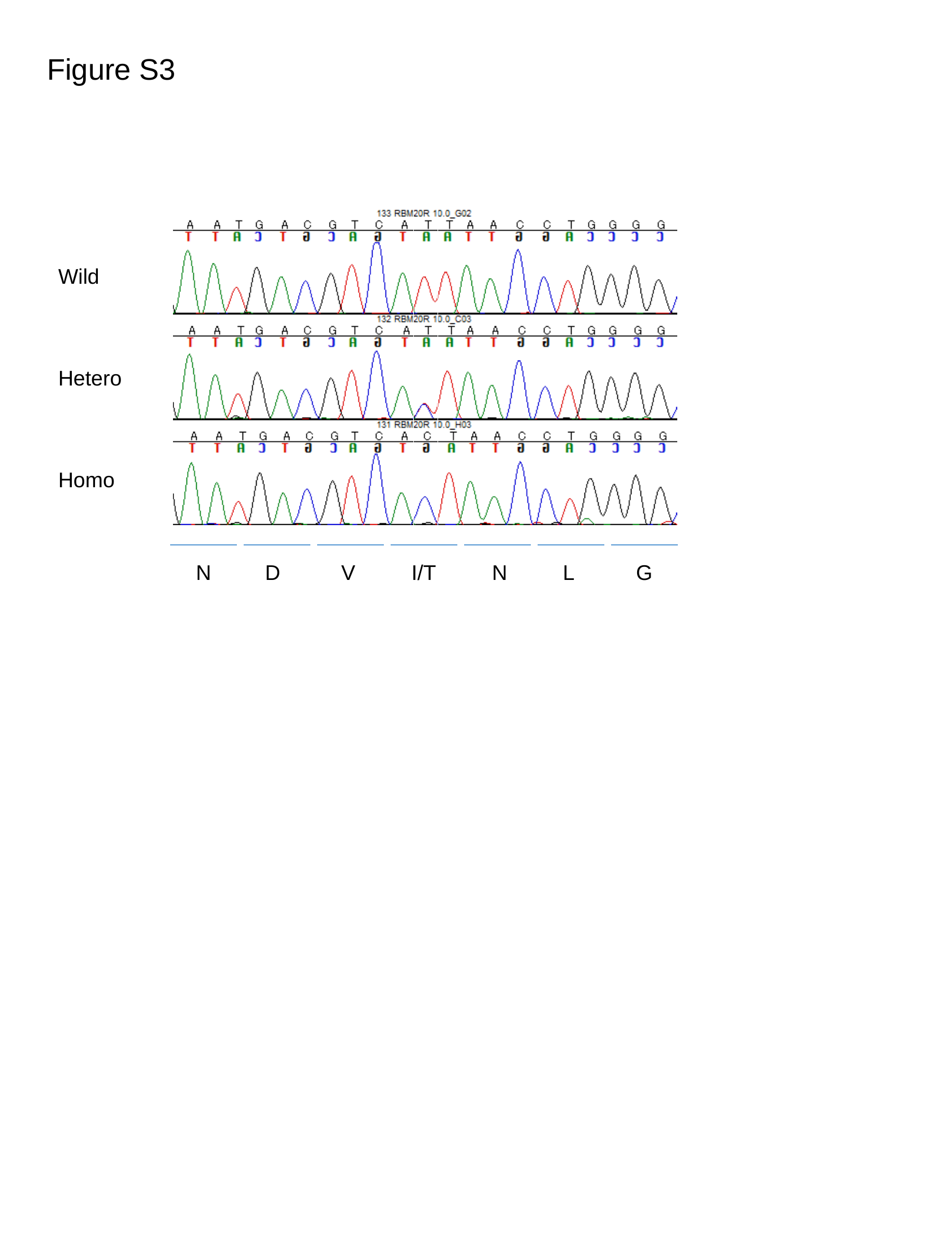

Figure S3
Wild
Hetero
Homo
N
D
V
I/T
N
L
G
